# Functional Expression of T-Type Ca^2+^ Channels in Spinal Motoneurons of the Adult Turtle

**DOI:** 10.1371/journal.pone.0108187

**Published:** 2014-09-25

**Authors:** Martha Canto-Bustos, Emanuel Loeza-Alcocer, Ricardo González-Ramírez, María A. Gandini, Rodolfo Delgado-Lezama, Ricardo Felix

**Affiliations:** 1 Department of Physiology, Biophysics and Neuroscience, Center for Research and Advanced Studies of the National Polytechnic Institute (Cinvestav-IPN), Mexico City, Mexico; 2 Department of Molecular Biology and Histocompatibility, “Dr. Manuel Gea González” General Hospital, Mexico City, Mexico; 3 Department of Cell Biology, Cinvestav-IPN, Mexico City, Mexico; University of Houston, United States of America

## Abstract

Voltage-gated Ca^2+^ (Ca_V_) channels are transmembrane proteins comprising three subfamilies named Ca_V_1, Ca_V_2 and Ca_V_3. The Ca_V_3 channel subfamily groups the low-voltage activated Ca^2+^ channels (LVA or T-type) a significant role in regulating neuronal excitability. Ca_V_3 channel activity may lead to the generation of complex patterns of action potential firing such as the postinhibitory rebound (PIR). In the adult spinal cord, these channels have been found in dorsal horn interneurons where they control physiological events near the resting potential and participate in determining excitability. In motoneurons, Ca_V_3 channels have been found during development, but their functional expression has not yet been reported in adult animals. Here, we show evidence for the presence of Ca_V_3 channel-mediated PIR in motoneurons of the adult turtle spinal cord. Our results indicate that Ni^2+^ and NNC55-0396, two antagonists of Ca_V_3 channel activity, inhibited PIR in the adult turtle spinal cord. Molecular biology and biochemical assays revealed the expression of the Ca_V_3.1 channel isotype and its localization in motoneurons. Together, these results provide evidence for the expression of Ca_V_3.1 channels in the spinal cord of adult animals and show also that these channels may contribute to determine the excitability of motoneurons.

## Introduction

Motoneurons are efferent neurons that originate in the spinal cord and synapse with muscle fibers to control muscle contraction [1]. In response to hyperpolarization, motoneurons may generate an action potential (AP) firing pattern known as postinhibitory rebound (PIR) when the membrane potential returns to its resting value [2]. During PIR a brief but strong hyperpolarizing input transiently increases neuronal firing rate to much higher levels compared to that prior to the inhibitory input [3], [4]. It is acknowledged that PIR may be mediated by the activation of two types of ion channels known as hyperpolarization-activated cyclic nucleotide-gated (HCN) and low voltage-activated (LVA) Ca^2+^ channels [3], [5], [6].

In addition to their contribution to the rebound firing of APs, HCN channels are the dominant molecular component of the hyperpolarization-activated current (*I*_h_) that play a major role in pacemaking activity [2], [6], [7], while LVA Ca^2+^ channels (also known as Ca_V_3.1, Ca_V_3.2 and Ca_V_3.3) can modulate neuronal excitability by opening in response to small membrane depolarization. In addition to promote rebound firing, Ca_V_3 channels regulate low-amplitude intrinsic neuronal oscillations, promote Ca^2+^ entry, and boost synaptic signals [5], [8], [9].

Ca^2+^ currents play an important role in modulating excitability and AP firing in motoneurons [1], [10], [11]. Initial work by Beam and colleagues using patch clamp recordings in cultured embryonic mouse and chick motoneurons [12], [13] and in developing (P9-P16) mouse spinal motoneurons [14] showed three components of the whole-cell Ca^2+^ current. Test potentials to −50 mV or greater elicited a LVA (T-type) current (*I*_T_), and test potentials to −20 mV or greater evoked two high-voltage activated (HVA) additional components, one transient and one sustained. Subsequent studies in embryonic rat spinal motoneurons showed that mibefradil (Ro 40-5967), a T-type channel blocker, caused a dose-dependent inhibition of inward Ca^2+^ currents [15]. In addition, motoneurons recorded during the first two weeks of postnatal development in the rat abducens nucleus exhibits a bursting discharge profile associated to the presence of prominent T- and H-type currents [16].

Examination of the ontogeny of Ca^2+^ currents in rat phrenic motoneurons and their role in electrical excitability during the embryonic and perinatal periods has shown changes in the expression of LVA and HVA channels. These changes include a decrease in the density of LVA and an increase in the density of HVA channels [12], [17], [18]. However, a subset of motoneurons in the P10–P13 age range not only expresses HVA but also LVA Ca^2+^ currents [14], [16], which is consistent with the expression of mRNAs encoding T-type channels (Ca_V_3.1 and Ca_V_3.2) in the adult rat spinal cord motoneurons [19]. Studying these currents is of particular interest because mice are able to bear weight and walk at this age and therefore can be considered to be motor-functionally mature [14].

Based on these findings, there is an ongoing debate regarding the presence of LVA (T-type) channels in motoneurons of adult animals. However, studies on the expression of these channels in mature motoneurons have been hampered by lack of suitable animal model systems. In the present study, we used an *in vitro* preparation of adult turtle spinal cord, a system much more resistant to hypoxia than the immature rodent spinal cord slide preparations, and show that the T-type Ca^2+^ channels participates in motoneurons PIR and modulate their excitability.

## Methods

### Spinal cord preparation

Adult turtles (*Trachemys scripta* spp) were anesthetized by hypothermia as described elsewhere [20] followed by intracardiac perfusion with a cold Ringer solution (NaCl 120 mM, KCl 5 mM, NaHCO_3_ 15 mM, MgCl_2_ 2 mM, CaCl_2_ 3 mM, and 20 mM glucose, saturated with 98% O_2_-2% CO_2_, pH 7.6) and decapitation. A laminectomy was performed to isolate the lumbar enlargement. Transverse spinal cord segments (∼2–3) mm were cut from the lumbar enlargement, in some cases in continuity with the ventral roots. Segments were placed in a recording chamber bathed with Ringer solution at room temperature (∼22°C). All experimental procedures were carried out in strict accordance with the recommendations of the Institutional Animal Care and Use Committee (CICUAL; Protocol Number: 239-05) and in accordance with the current Mexican norm for care and use of animal for scientific purposes (NOM-062-ZOO-1999). The Ethics Committee of the Center for Research and Advanced Studies of the National Polytechnic Institute (Cinvestav-IPN) approved this study. Animals were provided by the National Mexican Turtle Centre (Oaxaca, Mexico) with authorization (DGVS-03821/0907) of the Ministry of Environment and Natural Resources (Semarnat).

### Identification of motoneurons and electrophysiology

Recordings were made from the lumbar ventral horn (where motoneurons are located) identified under bright-field microscopy, using conventional intracellular recordings in the current clamp mode with an Axoclamp-2B amplifier (Axon Instruments) and electrodes filled with 0.8 M CH_3_COOK and 0.2 M KCl (25–35 MΩ). The bridge was balanced during routine recordings. Cells were classified as motoneurons if the input resistance was <80 MΩ, the AP presented fast and slow posthyperpolarizations and the firing pattern showed adaptation [4], [21]. In addition, 14 motoneurons were identified by antidromical stimulation of ventral roots [4], employing a suction electrode connected to a differential AC amplifier. Only motoneurons with resting membrane potential ≤−65 mV and with APs ≥80 mV were included in the study (*n* = 54). Of the total cells recorded, 40 exhibited low-threshold spiking and/or showed sensitivity to T-type channel antagonists. PIR was generated from a holding membrane potential (*V*_m_) of −61 to −58 mV applying 500 ms negative current pulses. Recordings were digitized (Digidata A/D 1322A, Axon Instruments), visualized using AxoScope software (Axon Instruments) and stored in the hard disk of a personal computer for off-line analysis.

### Data analysis

Spike amplitude was estimated as the maximal change in voltage membrane potential at rest to the peak of the spike. Measurements were made before and after *I*_h_ and *I*_T_ blockers application. Time to peak was measured from the end of the negative pulse to the peak of the spike. The amplitude of the PIR response was the average of 15 sweeps for each experimental condition per neuron. Rheobase was measured at the same membrane potential in both experimental conditions, and was estimated by increasing the amplitude of depolarizing square-wave current steps (500 ms duration) until a single spike was elicited. The values shown represent mean ± standard error for each experiment. The effects of the T-type channel antagonists on excitability were determined by the shift of the curve obtained by plotting the value of the depolarizing current *versus* the number of APs evoked. According to the Kolmogorov-Smirnov statistical test applied, data showed a normal distribution. Statistical significance was evaluated by either Student’s t test or one-way analysis of variance (ANOVA) followed by Tukey’s post hoc test for comparison of multiple means.

### Reverse transcription polymerase chain reaction (RT-PCR)

Immediately after dissection, total RNA was extracted from the lumbar enlargement of the spinal cord using TRIzol reagent (Invitrogen). For cDNA synthesis, total RNA samples (5 µg) were subjected to reverse transcription with 1 µl oligo-dT (500 µg/ml) and 1 µl (200 U) M-MLV RT enzyme (Invitrogen) in 20 µl of reaction mixture at 37°C for 1 h. cDNA amplification was carried out by PCR reaction in a total volume of 50 µl∶5 µl of cDNA, 1× PCR buffer (20 mM Tris-HCl, 50 mM KCl, pH 8.4), 0.2 mM of each deoxynucleotide triphosphate, 1.5 mM MgCl_2_, 0.5 µM of each primer and 2.5 U of Taq DNA polymerase (Invitrogen) on a PCR thermal cycler. PCR primers were designed to amplify conserved regions of Ca_V_3 channels. For Ca_V_3.1, the forward primer sequence was 5′-cacttgtgcaccagccacta-3′ and the reverse primer sequence was 5′-aggtccaaagagctccac-3′; and for actin the forward primer sequence was 5′-aagatgacccagatcatgtt-3′ and the reverse primer sequence was 5′-gagtacttgcgctcaggagg-3′. The reaction was performed as follows: 30 cycles of 95°C for 45 s, 55°C for 30 s and 72°C for 1 min. PCR products were electrophoresed on 1% agarose gels, stained with ethidium bromide and analyzed under ultraviolet light. The identity of the amplicons was confirmed by automated sequencing.

### Western blot

Immediately after dissection, the lumbar enlargement of the adult turtle spinal cord was homogenized in lysis buffer containing 50 mM Tris-HCl pH 8.0, 150 mM NaCl, 0.5 mM phenylmethylsulfonyl fluoride, 1% NP-40 and Complete 1× (Roche). Cell lysates were then centrifuged at 12,000×g for 2 min. Protein concentration was determined by the bicinchoninic acid method. One hundred µg of proteins were mixed with Laemmli sample buffer and boiled for 5 min. Proteins were separated on 8% SDS-PAGE and transferred onto nitrocelulose membranes (Biorad). Membranes were blocked for 1 h at room temperature in TBS-T (150 mM NaCl, 10 mM Tris-HCl, pH 8, 0.05% Tween 20) containing 5% low-fat dried milk and then incubated overnight at 4°C with the anti-Ca_V_3.1 antibody (Alomone). After three washes in TBS-T, membranes were incubated with a horseradish peroxidase conjugated secondary antibody (Jackson ImmunoResearch). Protein bands were detected using an enhanced chemiluminescence system (Millipore).

### Spinal cord immunostaining

Immediately after dissection, spinal cord segments (2–3 mm) were fixed in 4% paraformaldehyde in PBS for 24 h as previously described [22]. After fixation, samples were cryoprotected by suspending them overnight in PBS containing 30% sucrose at 4°C and then sliced using a cryotome (30 µm). Free-floating tissue sections were made permeable (0.3% Triton X-100 in PBS) for 10 min and blocked in PBS blocking solution 1 (1% gelatin and 10% FBS) for 30 min at room temperature. Spinal cord sections (30 µm) were first incubated with an anti-ChAT primary antibody (24 h at 4°C, 1∶50, Millipore) and then revealed using a FITC goat anti-rabbit secondary antibody (2 h at room temperature, 1∶200, Jackson ImmunoResearch). Subsequently, sections were incubated with an anti-Ca_V_3.1 primary antibody (24 h at 4°C, Santa Cruz Biotechnology; 1∶100 dilution), and then exposed 1 h to the secondary antibody (1∶200; Dylight 549-conjugated anti-rabbit IgG; Jackson ImmunoResearch). Samples were examined using confocal laser scanning microscopy (Leica TCS SP2). Images were obtained with the filter set for Dylight 549 using the 40× oil immersion plan apochromatic objective (NA 0.8).

### Drugs

Functional assays were performed in the present of 6-Cyano-7-nitroquinoxaline-2,3-dione (CNQX; 20 µM), strychnine (2 µM) and (2R)-amino-5-phosphonovaleric acid (APV; 40 µM). During electrophysiological recordings NNC55-0396 or Ni^2+^ was used to block *I*_T_ and ZD7288 was used to block *I*_h_. All drugs were purchased from Sigma-Aldrich.

## Results

**Figure 1:**
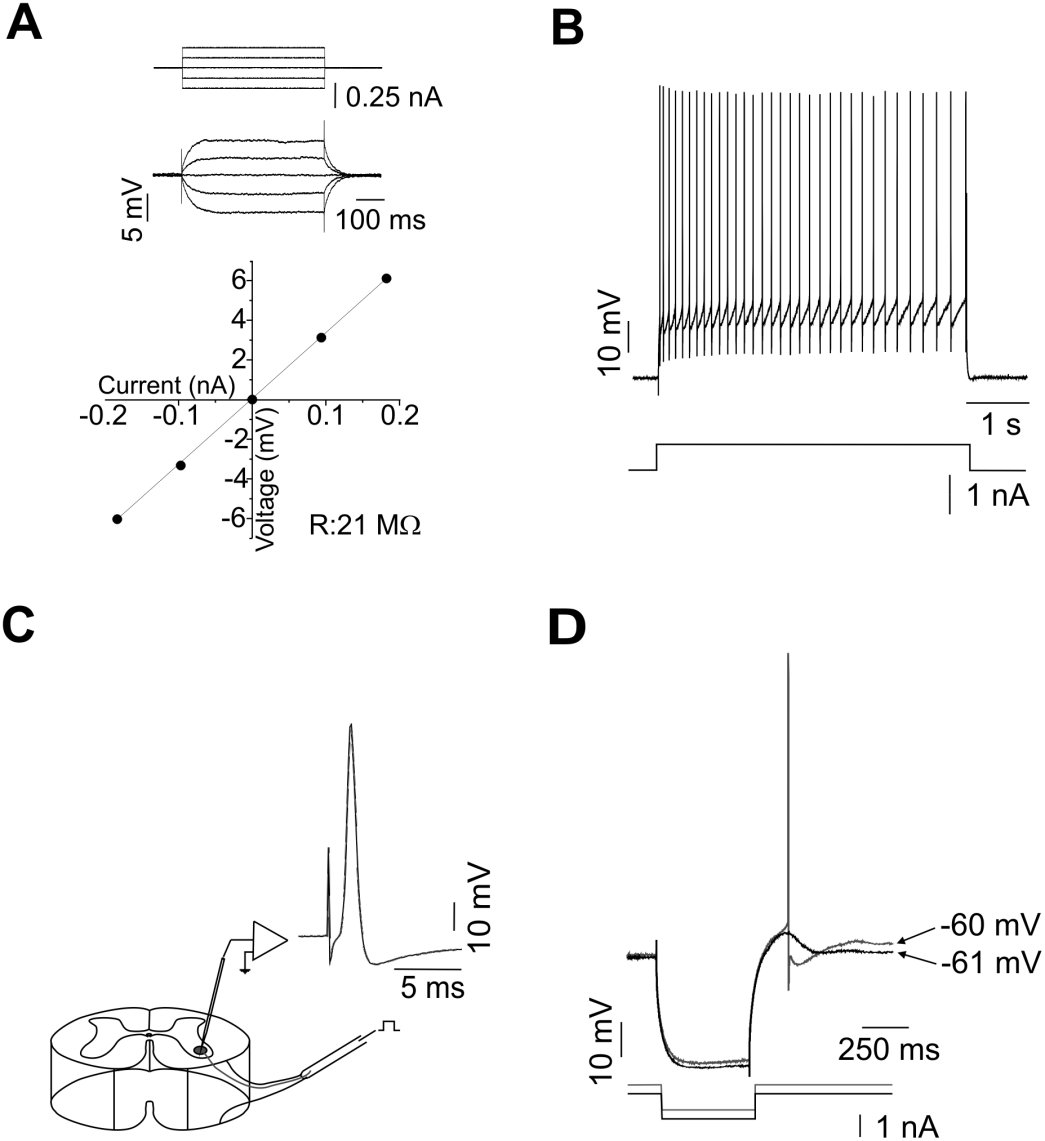
Electrophysiological characterization of adult turtle motoneurons. A) Measurement of the membrane input resistance (*R*_m_) voltage-dependency. *R*_m_ was measured within ±10 mV around *V*_m_. The upper panel shows typical voltage deflections of an adult turtle motoneuron. The lower panel shows *R*_m_ values estimated from plateau values of each voltage trace (symbols) as a function of the current pulse by calculating the slope of the linear part of the *I−V* curve. *R*_m_ was in a range of 9–76 MΩ. B) Spike trains elicited in an adult turtle motoneuron by current injection in the control condition. Note the spike frequency adaptation. C) Typical antidromical AP generated in a motoneuron in response to ventral root stimulation. D) Superimposed traces of PIR responses evoked by hyperpolarizing current pulses at two different *V*_m_ values as indicated.

The results described here were obtained from 54 cells in which a stable resting potential (*V*_m_) could be maintained for up to 3 h. The intracellular recordings were made in bridge mode and voltage excursions were obtained by applying a bias current (−0.5 to −2 nA). Only cells with *V*_m_ values between −65 to −79 mV (−69±3.5 mV) were considered for pharmacological analyses. It is worth noticing here that previous studies have shown that the electrophysiological properties of turtle motoneurons are similar to those reported for mammals [4]. Hence, the whole AP usually lasted for 0.7 to 1.4 ms (1.04±0.15 ms) with a rise time in the range of 0.5 to 1.0 ms (0.72±0.11 ms), and the spike amplitude variation was between 80 and 107 mV (93.3±8.1 mV). The input resistance of the ventral horn neurons recorded was in the range of 9 to 76 MΩ (22.8±11.6 MΩ), and the time constant varied between 8.2 and 45 ms (23.1±9.1 ms; Figure 1A). In addition, recordings showed the typical adaptation pattern observed in motoneurons in response to depolarizing current pulses (Figure 1B) as previously reported [4], [21]. Only cells displaying the distinctive properties of motoneurons [4] were selected for analysis. Of these cells, 14 were further identified by the antidromical stimulation of ventral roots (Figure 1C).

The postinhibitory rebound (PIR) response occurs at the termination of a hyperpolarizing event in a voltage dependent manner at membrane potentials near the spike threshold as has been observed in neuronal and non-neuronal cells [2]. In the preparation of the adult turtle spinal cord, the rebound responses were induced in motoneurons at different *V*_m_ levels by applying rectangular hyperpolarizing current pulses and bias current. When *V*_m_ was >−60 mV, PIR responses were strong enough to generate APs in a motoneurons identified by antidromic stimulation (Figure 1D), as reported previously [4]. At a *V*_m_ just below the threshold for AP firing, the PIR amplitude was measured at peak with the *V*_m_ level before the stimulus pulse taken as the baseline. In this condition, the maximal PIR amplitude observed was ∼7 mV (3.7±1.4 mV; *n* = 30).

**Figure 2:**
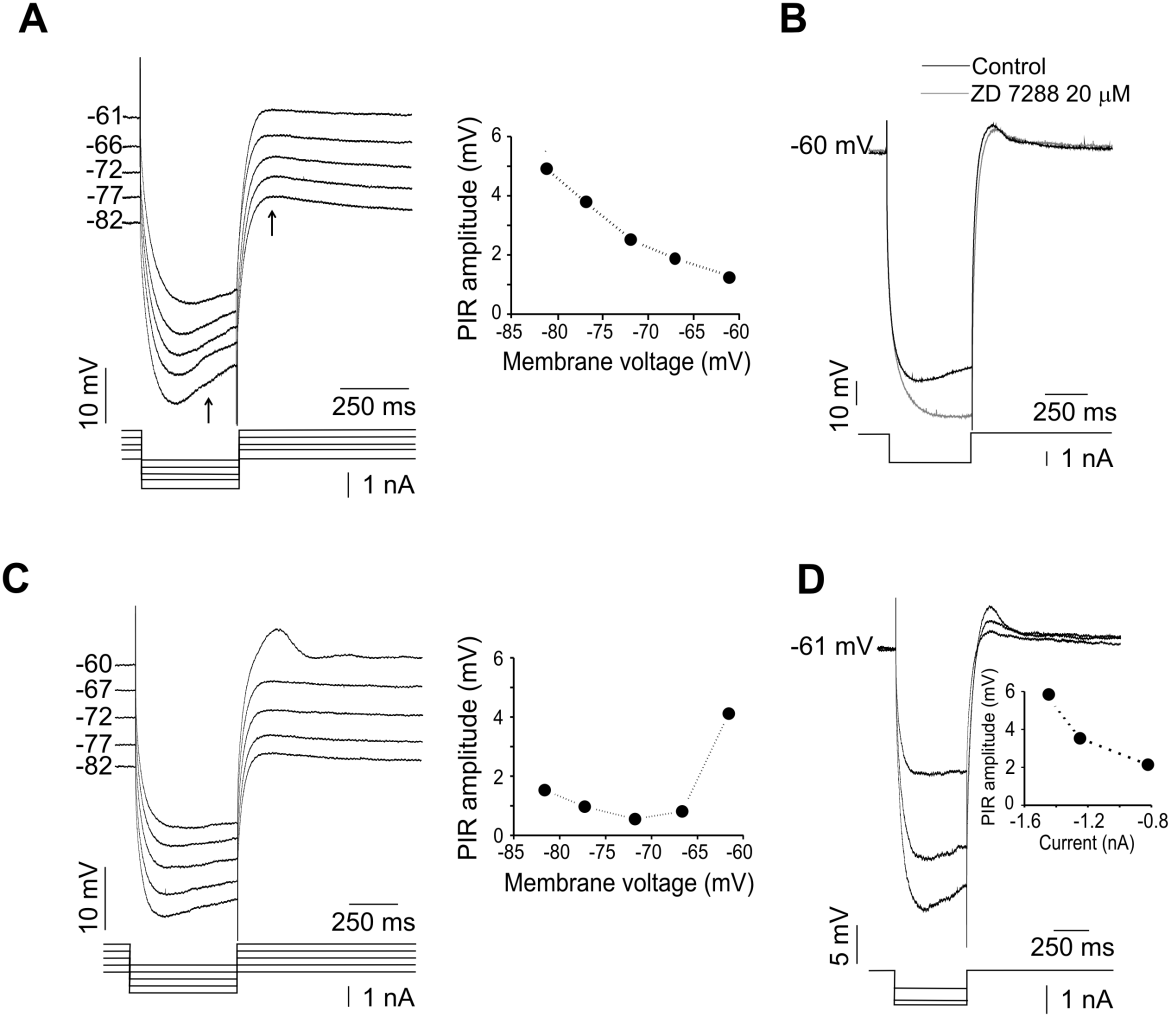
Two types of postinhibitory rebound responses (PIR) in adult turtle motoneurons. A) Rebound responses evoked by hyperpolarizing current pulses with the same intensity at different *V*_m_ values. For clarity, in the voltage traces the PIR and the voltage sag components are indicated by arrows. The right panel shows PIR amplitudes as a function of *V*_m_. PIR amplitude is larger at more negative *V*_m_ values which is suggestive of HCN channel activation. B) A residual PIR persists after the application of an *I*_h_ current blocker (ZD7288). Note that the voltage sag was eliminated by the *I*_h_ antagonist. C) Rebound responses evoked by hyperpolarizing current pulses at different *V*_m_ values. The right panel shows PIR amplitudes as a function of *V*_m_. PIR amplitude is similar in the voltage range of −82 to −67 mV but is bigger at −61 mV suggesting a recruitment of T-type channels. D) PIR amplitude increases with pulse intensity. Rebound responses were evoked by hyperpolarizing current pulses of increasing amplitude (inset) from a *V*_m_ of −61 mV.

The sag and postdepolarization phases were dependent on *V_m_*. In some cases (*n* = 6), in response to the same hyperpolarizing current pulse, PIR showed a decrease in amplitude as *V*_m_ was increased. This could be explained by the activation of HCN channels [6]. In the example shown in Figure 2A, the voltage excursion from −82 to −61 mV caused a decrease in HCN channels activation and consequently a reduction in PIR amplitude. This interpretation is supported by the presence of a voltage sag during the hyperpolarizing pulses which is typically caused by the activation of the *I*_h_ current through HCN channels. Therefore, the contribution of the *I*_h_ current to the rebound response was investigated. In the presence of an *I*_h_ blocker, ZD7288 (20 µM), PIR amplitude was partially reduced to 66±15% in five neurons held at a *V*_m_ of −60 mV (Figs. 2B; and 3C). This reduction most likely results from blockade of HCN channels.

On the other hand, in a subset of cells tested (5 out of 11), the PIR amplitude was similar within the voltage excursion ranging from −82 to −67. However, an increase in PIR amplitude (∼4 mV) was observed at a *V*_m_ of −60 mV (Figure 2C). This effect may be associated with a recruitment of T-type channels that are first deinactivated by hyperpolarization and then activated during the repolarization period. In eleven motoneurons, the same response was observed when the magnitude of the hyperpolarization was bigger with current pulses of increasing amplitude (−1.5 to −0.4 nA; Figure 2D). The combined results of partial block with ZD7288 and increased PIR responses with negative current pulses suggested the presence of both HCN and T-type channels in motoneurons from the spinal cord of the adult turtle. Therefore we next sought to determine whether the postinhibitory rebound is modulated by targeting T-type channels.

**Figure 3:**
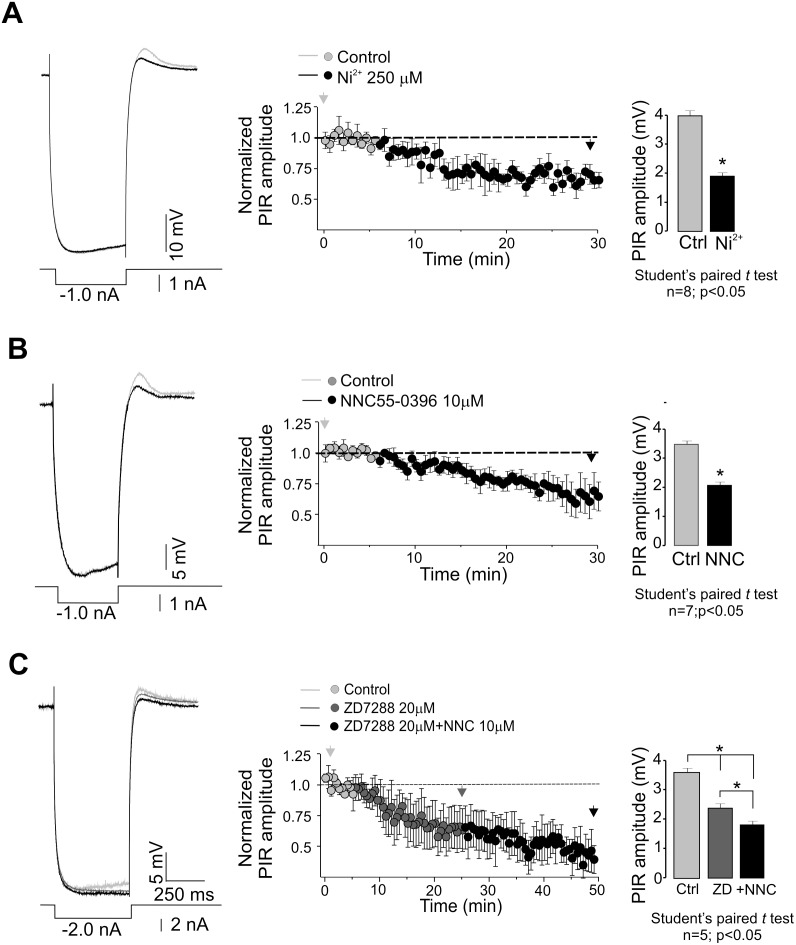
Contribution of T-type channels to PIR in adult turtle motoneurons. A) Inhibition of rebound depolarization by Ni^2+^. PIR responses were elicited by hyperpolarizing pulses from a *V*_m_ of −58 mV (left panel). Average time courses of the normalized peak amplitude of PIR in control conditions and after Ni^2+^ application are shown in the mid panel. Arrow heads denote indicate the time at which the data were analyzed. The bar chart in the right panel shows the comparison of PIR amplitude before and after Ni^2+^ application. B) Block of rebound depolarization by NNC55-0396. PIR responses were evoked by hyperpolarizing pulses from a *V*_m_ of −60 mV (left panel). Average time courses of the normalized peak amplitude of PIR in control conditions and after NNC55-0396 application are shown in the mid panel. Arrow heads denote indicate the time at which the data were analyzed. The bar chart at the right shows the comparison of PIR amplitude before and after drug application. C) Sequential application of ZD7288 and NNC55-0396 significantly inhibited postinhibitory rebound, further demonstrating the contribution of HCN and T-type channels to PIR. The average time courses of the normalized peak amplitude of PIR in control conditions and after drug application are shown in the mid panel. Arrow heads denote indicate the time at which the data were analyzed.

The possible contribution of T-type channels to the postinhibitory rebound was investigated using Ni^2+^ and NNC55-0396. Although Ni^2+^ may inhibit both LVA and HVA Ca^2+^ channels at high concentrations, it has much higher affinity toward LVA channels [5], [23]. Thus, the quantitative effects of Ni^2+^ and NNC55-0396, a more selective LVA channel antagonist, may provide important information on T-type channel functional expression in the adult turtle preparation. During pharmacological experiments, *V*_m_ was kept constant at the same level in control recordings and after drug applications. Left panel in Figure 3A shows an example of a cell in which the PIR amplitude was substantially decreased by Ni^2+^ (250 µM). This inhibitory effect 48±12% was seen in 8 cells tested (Figure 3A; right panel). In this case, the sag response was absent indicating that *I*_h_ may not contribute to PIR. The possible contribution of T-type channels to the rebound response was also investigated using NCC55-0396, a mibefradil derivative that has been reported to block T-type channels [24]. The amplitude of the rebound depolarization was decreased to ∼59±10% of the control mean value by NCC55-0396 application (n = 7), as illustrated in Figure 3B for a representative motoneuron antidromically identified.

The results summarized in Figs. 2 and 3 suggested that in addition to the T-type Ca^2+^ current (*I*_T_), the hyperpolarization-activated current (*I*_h_) may also contribute to the PIR in some recorded cells. The results shown in Figure 3C confirm that this is the case. The PIR amplitude was significantly decreased (∼30%) after application of 20 µM of the *I*_h_ antagonist ZD7288 (4-Ethylphenylamino-1,2-dimethyl-6-methylaminopyrimidinium chloride). Interestingly, the remaining PIR after the application of ZD7288 was also decreased by application of the T-type Ca^2+^ channel NCC55-0396 in 5 motoneurons (Figure 3C). The combined results with Ni^2+^ and NNC55-0396 application suggested that T-type channels significantly contribute to the postinhibitory rebound responses observed in 40 out of 54 motoneurons from the spinal cord of the adult turtle.

**Figure 4:**
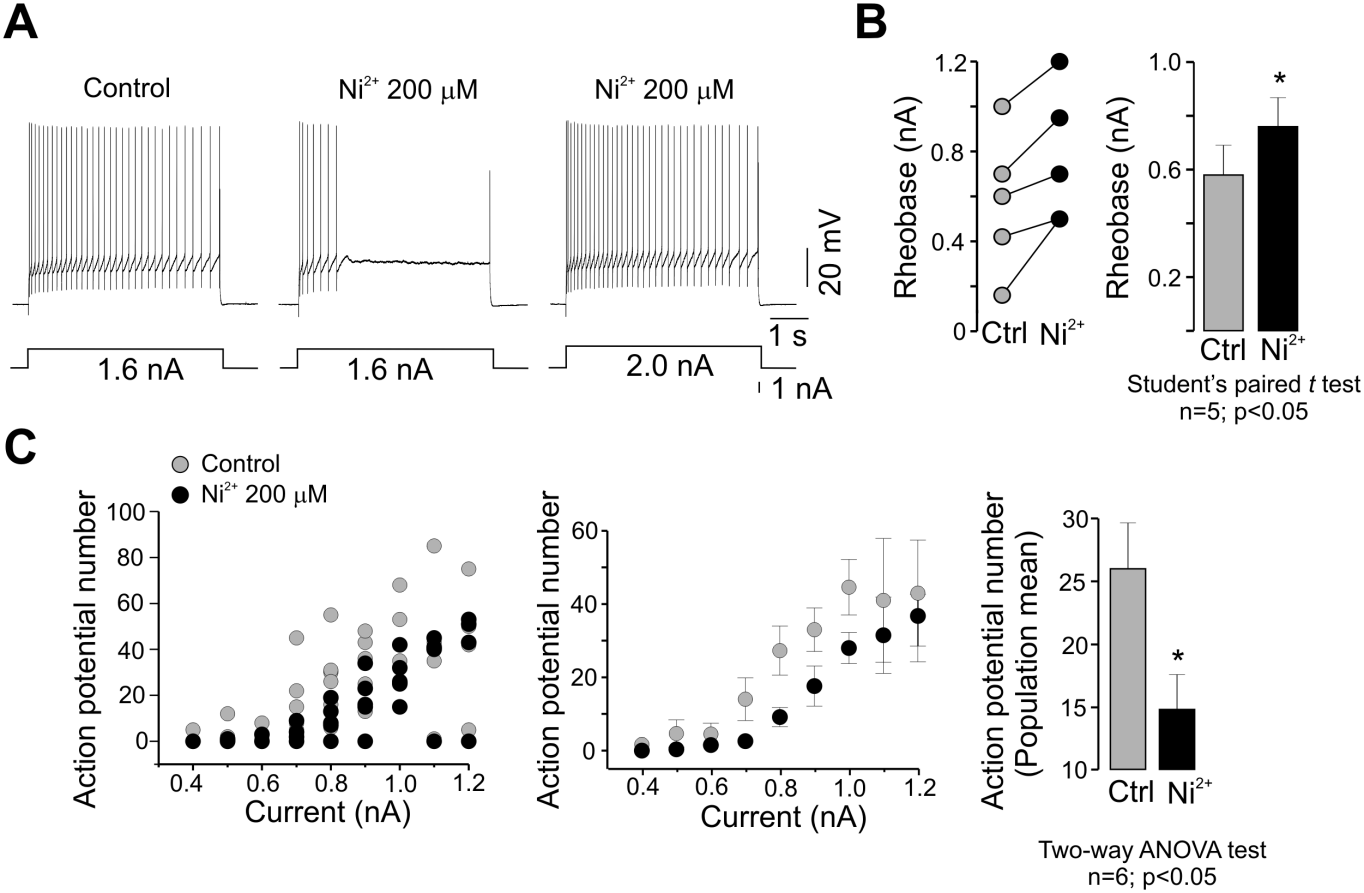
The T-type channel antagonist Ni^2+^ decreases adult turtle motoneuron excitability. A) Effect of Ni^2+^ on the AP firing rate. AP firing was elicited by applying a depolarizing current step of 1.6 nA (left panel). Note that the addition of Ni^2+^ greatly prevented AP firing (middle panel; *n* = 7). Stronger current injections restored AP bursts in the recorded cells (right panel). B) Comparison of the rheobase values in individual motoneurons before (Ctl) and after Ni^2+^ application. The bar chart summarizes the comparison of mean values in both experimental conditions. C) Comparison of the AP number as a function of current in individual (left panel) and grouped cells (middle panel) in the absence (Control) and the presence of Ni^2+^ as indicated. The bar chart compares the mean values in both conditions (right panel).

**Figure 5:**
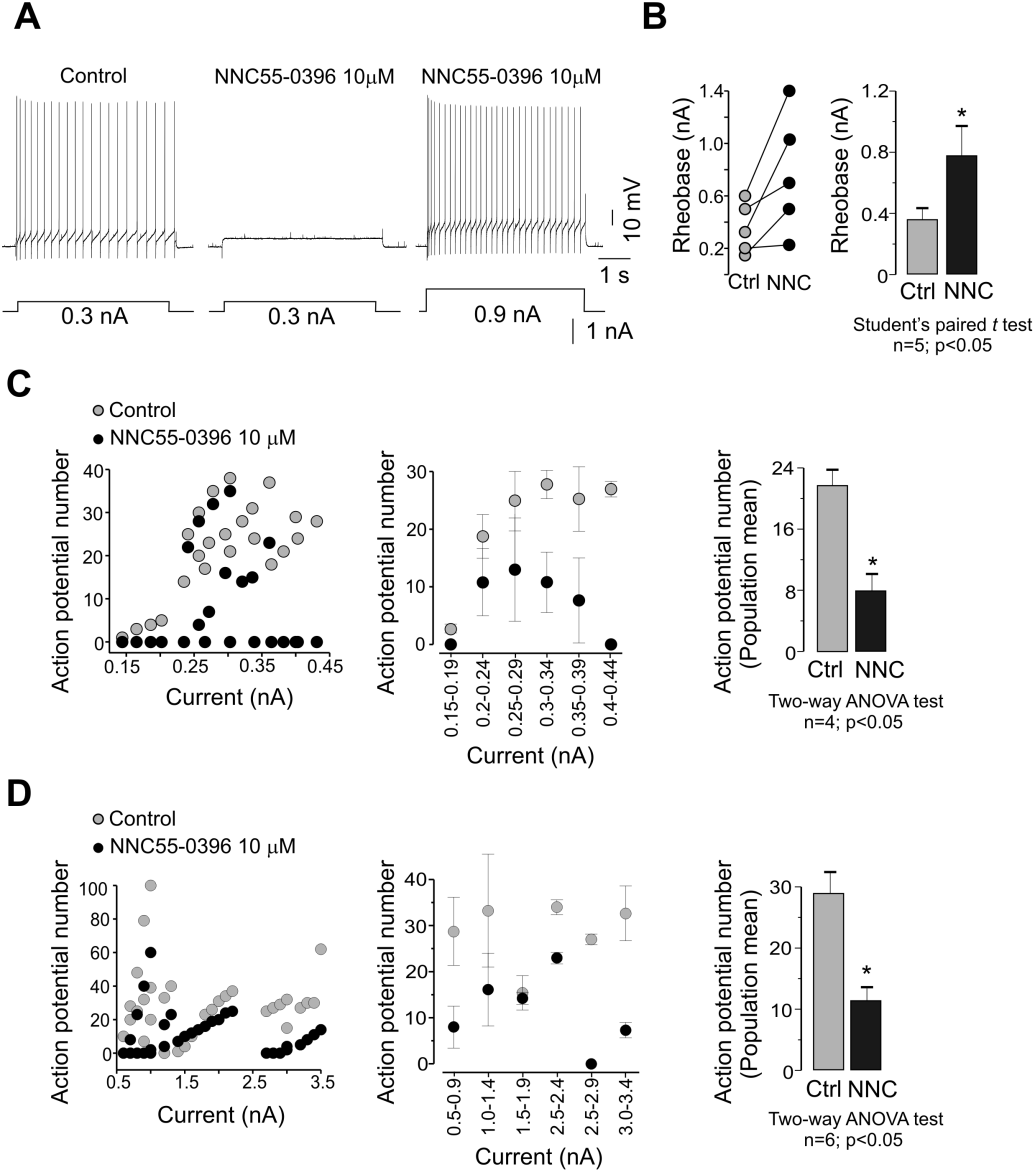
The T-type channel antagonist NNC55-0396 reduces adult turtle motoneuron excitability. A) Effect of NNC55-0396 on the AP firing rate elicited by applying a depolarizing current step of 0.3 nA (left panel). Note that NNC55-0396 application abolished AP firing (middle panel; *n* = 10). Stronger current injections restored AP bursts (right panel). B) Comparison of the rheobase values in individual motoneurons before (Ctrl) and after drug application. C) Comparison of the AP number as a function of current in the absence (Ctrl) and the presence of NNC55-0396 in the 0.15–0.44 nA range. D) Comparison of the AP number as a function of current in the absence and the presence of NNC55-0396 in the 0.5–3.4 nA range. The bar chart in C and D compare the mean values in both control and experimental conditions.

T-type channels have been identified in many central neurons and in peripheral sensory neurons. Given that these channels are available for opening only from very negative membrane potentials they are ideally suited for regulating neuronal excitability [3], [8], [9]. Therefore, we next decided to investigate the role of T-type channels in motoneuron excitability, by evaluating the effects of Ni^2+^ and NNC55-0396 on firing frequency of APs generated by the application of positive current intracellular pulses. Figures 4 and 5 show that spiking in motoneurons from the adult turtle spinal cord antidromically identified was compromised after the application of the two T-type channel antagonists.

Addition of 200 µM Ni^2+^ to the bath solution decreased the action potential firing in 7 neurons tested, and it was necessary stronger current injections to restore AP bursts (Figure 4A). As a result, the AP number-current curves for individual and grouped cells were shifted to the right (Figure 4C). Furthermore, generation of APs was prevented in some motoneurons when Ni^2+^ was applied (Figure 4A middle panel), causing an increase in the rheobase (Figure 4B). Similarly, addition of 10 µM NNC55-0396 decreased the AP firing in 10 neurons, and it was necessary also stronger current injections to restore AP bursting (Figure 5A). As in the case of the experiments performed in the presence of Ni^2+^, the AP number-current curve was shifted to the right (Figs. 5C and 5D). For clarity, these curves were separated in two groups depending on the magnitude of the current pulse applied. This was done because the variability in the input resistance of the cells incubated with NNC55-0396 was broader than those used in the Ni^2+^ test. Nevertheless, the curves were shifted to the right (Figs. 5C and 5D). Likewise, generation of APs was also prevented in some motoneurons when NNC55-0396 was used (Figs. 5A middle panel and 5C left panel), causing an increase in the rheobase (Figure 5B). Taken together, these data suggest that T-type channels may play a role in the generation of rebound spikes and the control of excitability in motoneurons from the adult turtle spinal cord.

**Figure 6:**
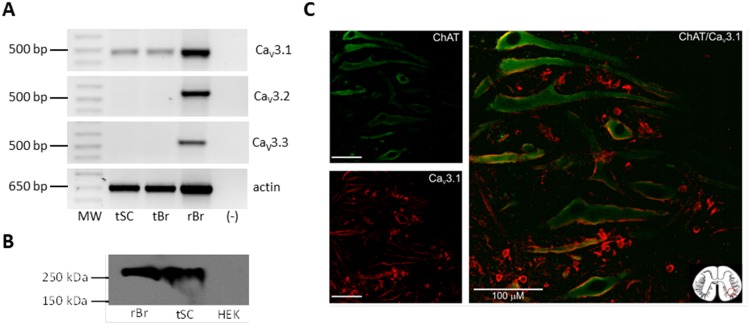
Ca_V_3.1 channel expression in adult turtle spinal cord. A) RNA was extracted from the adult turtle spinal cord (tSC) and rat brain (rBr), used as a positive control, and subjected to RT-PCR with specific primers. Molecular weight markers are on the left, and (-) denotes negative control without RT enzyme. B) Proteins extracted from the turtle spinal cord (tSC) and rat brain (rB; used as a positive control) were subjected to Western-blot using anti-Ca_V_3.1 antibodies. A ∼250 KDa band was present both in the TSc lane and the positive control. C) Representative confocal micrographs from adult turtle spinal cord slices immunostained with choline acetyltransferase (ChaT; a marker for motoneurons) shown in the left upper panel (green) and Ca_V_3.1 antibodies shown in the left lower panel (red), suggesting co-localization of both proteins (right panel). Scale bar = 100 µm.

Previous studies have identified three main subtypes of T-type channels named Ca_V_3.1, Ca_V_3.2 and Ca_V_3.3 [8]. Therefore, to identify the T-type channel isotype(s) that possible mediate PIR responses in the motoneurons from the adult turtle spinal cord, we then searched for Ca_V_3 channel mRNAs expression in the lumbar enlargement. To this end, specific primers directed toward conserved regions in the Ca_V_3 channel sequences were designed, and total RNA samples were analyzed by RT-PCR. The results of this analysis showed the presence of a band of the expected size (455 bp) corresponding to the Ca_V_3.1 channel (Figure 6A). The primers used in these experiments did not allow to amplify the mRNA for the Ca_V_3.2 and Ca_V_3.3 isotypes in the turtle spinal cord, however their expression cannot be ruled out given that the primes used were designed based on mammalian sequences. The identity of the Ca_V_3.1 amplicon was confirmed by comparison to the positive control obtained from a rat brain RNA sample and by automated sequencing (Figure 6A; Figure S1). Conventional multiple sequence alignment of turtle spinal cord Ca_V_3.1 isotype revealed >90% overall identity within different species (Figure S2). The sequence reported in this paper is being deposited in the GenBank database.

The second line of experimental evidence supporting the expression of Ca_V_3.1 channels in the adult turtle spinal cord was obtained using antibodies. Western blot analyses of rat brain and adult turtle spinal cord homogenates with Ca_V_3.1 channel antibodies showed a prominent band (Figure 6B) of the expected mass for the full-length Ca_V_3.1 polypeptide (∼250 kDa). No signal was observed in membranes from HEK-293 cells used as a negative control. Last, immunohistochemical staining was performed on transverse slices of the turtle lumbar spinal cord. As can be seen in Figure 6C, Ca_V_3.1 immunostaining was prominent in cells co-expressing choline acetyltransferase immunoreactivity (a marker for motoneurons), where signal was dispersedly distributed in the somata and proximal dendrites, sparing the nucleus.

It should be noted, however, that the primary antibody used in these experiments recognizes the mammalian Ca_V_3.1 channels, but it has not previously tested in turtle tissues. Therefore, the possibility exists that such antibody might be cross-reacting with a Cav3-like molecule in the spinal cord of the turtle. However, we consider that the antibody is more likely interacting with the Ca_V_3.1 protein given that the preincubation of the primary antibody with the antigenic peptide used to raise the Ca_V_3.1 abolished any signal (Figure S3). In addition, no labeling was seen in the absence of the primary antibody.

## Discussion

Previous studies in rat spinal motoneurons have revealed the presence of a significant afterdepolarization following the action potential of embryonic cells, which is not the case in the adult motoneurons of the turtle. The reason for this discrepancy is not fully understand, but it may lie in the dynamic spatial and temporal regulation of membrane ion channel gene expression. In this regard, it should be noted that several conductances can be associated to afterhyperpolarization in motoneurons such as delayed rectifier, transient outward, Ca^2+^-activated K^+^, hyperpolarization-activated, voltage-activated Ca^2+^ and leak currents, among others [1]. Interestingly, the expression of these channels appears to under a tight temporal control during ontogeny or restricted to certain motoneuron subpopulations [1].

On the other hand, postinhibitory rebound response is a commonly observed feature in many species from crustaceans to vertebrates [2]. Though the mechanisms may vary in different neuronal types, several ionic fluxes may be of critical importance in the development of PIR. These include the activation of hyperpolarization-activated cyclic nucleotide-gated or T- and L-type Ca^2+^ channels as well as the activation of Ca^2+^-activated Cl^−^ and inward rectifying currents [6], [25], [26]. Among these mechanisms, the presence and activity of the HCN and T-type channels may explain the results we obtained in the adult turtle motoneurons. The contribution of these channels was evidenced by using ZD7288 which blocked HCN channels and reduced partially the PIR response without effecting T-type channel activity [27], followed by the application of NNC55-0396 a selective T-type channel antagonist which caused an additional inhibition in the PIR response.

It is worth mentioning that NNC55-0396 is a derivate of mibefradil which is known to block both T-type and HVA Ca^2+^ channels. However, mibefradil effect on HVA channels is not direct but instead involves cell permeation and hydrolysis to an active metabolite that acts from the cytoplasmic side of the membrane [28]. In contrast, NNC55-0396 is not hydrolyzed to an active metabolite and does not block HVA currents [24]. Thus, NNC55-0396 is a selective inhibitor for T-type Ca^2+^ channels. Although T-type channel antagonist might have different specificity in turtle tissue, it should be noted, however, that NNC55-0396 may inhibit T-type channel activity in several biological preparations. Hence, the drug has been shown to block both recombinant Ca_V_3 channels expressed in HEK-293 cells [23], [24], [29] as well as native T-type channels expressed in 3T3-L1 preadipocytes [30], as well as the Ca^2+^ transients associated to T-type channel activity in human myometrium [31] and pregnant rat uterine smooth muscle [32]. NNC55-0396 also blocks T-type Ca^2+^ channel-mediated rebound firing cerebellar neurons [33] and the T-type channel-mediated response to nerve stimulation in rat vas deferens [34].

These results are in agreement with studies that have implicated *I*_T_ and *I*_h_ as primary contributing factors in controlling the frequency and latency of rebound responses [4], [5], [35]. We found that *I*_T_ was responsible for ∼50% of the PIR response activated by a hyperpolarization. Such a conclusion is based on two lines of evidence: i) PIR was found to be blocked by low concentrations of Ni^2+^ (200 µM), which does not block L-type Ca^2+^ channels in dorsal horn neurons [5], and ii) the current underlying PIR was also blocked by NNC55-0396, a selective antagonist of T-type channels.

As mentioned earlier, the developmental profile of *I*_T_ in motoneurons is quite complex [10]. Though transient expression of T-type channels has been reported in brainstem and spinal motoneurons [13], [15], [36], in spinal cord motoneurons these channels decrease after P7-P8 [10], [13], nevertheless it does not completely disappear. Interestingly, our biochemical and molecular studies showed the expression of the Ca_V_3.1 channels in the adult turtle spinal cord. Further immunofluorescence studies revealed that these channels were indeed present in motoneurons. Although the presence of Ca_V_3 channel mRNAs has been shown previously in adult rat spinal motoneurons [19], to our knowledge this is the first report of the Ca_V_3.1 protein in mature motoneurons.

It is worth noting also that the rebound postinhibitory potential mediated by T-type channels has been observed in motoneurons of the abducens nucleus after P7 [16]. Though these studies have not been performed in functionally mature motoneurons, its firing properties resemble that recorded in the same neurons in the adult rat and cat [37], [38]. As mentioned above, at the adult stage two T-type channel transcripts (Ca_V_3.1 and Ca_V_3.2) are expressed in rat motoneurons [19]. Thus, it is conceivable that the *I*_T_ may be maintained in mammalian adult motoneurons, as we found in the adult turtle.

Our results evidenced an important role for T-type channels in determining motoneuron excitability in the adult turtle. Using Ni^2+^ and NNC55-0396 we found that the *I*_T_ is one of the major determinants of AP generation. The physiological relevance of these results is that the increased recruitment of T-type channels with hyperpolarization confers robustness to depolarization associated with diverse inputs to further recruit these deinactivated channels, resulting in a transient Ca^2+^ current that increases the firing probability. These results are in agreement with diverse studies showing that different Ca_V_3 channels play crucial roles in AP firing in inferior olivary neurons, Purkinje cells and thalamic neurons [39]–[42]. Likewise, the inhibition of T-type channels by G protein activation decreases the excitability of small dorsal root ganglion neurons [25]. It is worth mentioning, however, that *I*_T_ may represent a primary, but not the exclusive, ionic factor responsible for generating the increase in spike frequency during the rebound response in motoneurons of the adult turtle spinal cord. Our results indicate that the rebound response must incorporate additional factors beyond *I*_T_ as an excitatory influence. Indeed, our own assessment revealed an additional influence of *I*_h_ on the posthinibitory rebound response in these cells.

The results of our work are of importance in determining the role of T-type channels for a better understanding of the mechanisms underlying both the modulation of single neurons and the overall operation of the spinal locomotor networks. Indeed, activation of *I*_h_ upon hyperpolarization beyond resting potential together with *I*_T_ might represent an advantageous mechanism for determining integrative behavior near rest and might provide the pacemaker depolarization during generation of rhythmic-oscillatory activity.

## Supporting Information

Figure S1 ***In silico*** **analysis of the turtle Ca_V_3.1 sequence. Alignment of the Ca_V_3.1 partial sequence (455 nucleotides) from the spinal cord of the adult turtle with different species as indicated.** Sequences were downloaded from GenBank and were aligned using the Vector NTI sequence alignment software (Invitrogen). Red highlights residues that are identical among different species, blue indicates residues shared among species and black the residues that are unique.
(TIF)

Figure S2 **Comparison of the level of similarity among all Ca_V_3.1 sequences shown in Figure S1.**
(TIF)

Figure S3 **Specificity of the Ca_V_3.1 immunostaining in neurons of the adult turtle spinal cord.** Images of transversal sections of the lumbar region are shown. A) Ventral horn Ca_V_3.1 immunoreactivity (green); neuronal nuclei are stained with DAPI (blue). B) The anti-Ca_V_3.1 antibody was pre-incubated with an excess of its antigenic peptide and added to the sample. C) Bright field image of panel B. Scale bar 50 µm.
(TIF)
