# Supplementary figures and images for: Functional Expression of T-Type Ca2+ Channels in Spinal Motoneurons of the Adult Turtle
Source: PLoS One. 2014 Sep 25;9(9):e108187. doi: 10.1371/journal.pone.0108187 (PMC4177857; doi:10.1371/journal.pone.0108187)

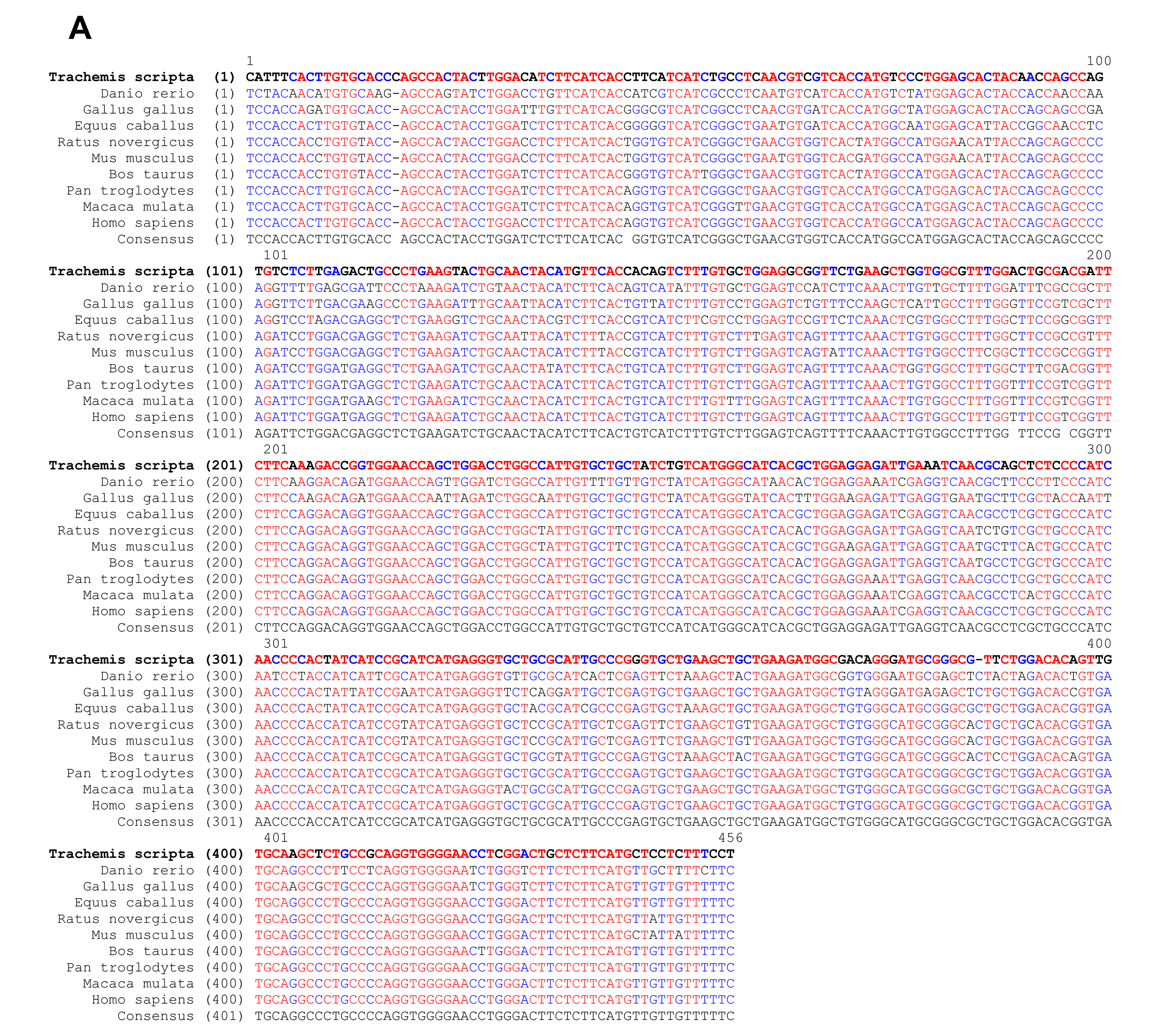

Supplement: Figure S1 — In silico analysis of the turtle CaV3.1 sequence. Alignment of the CaV3.1 partial sequence (455 nucleotides) from the spinal cord of the adult turtle with different species as indicated. Sequences were downloaded from GenBank and were aligned using the Vector NTI sequence alignment software (Invitrogen). Red highlights residues that are identical among different species, blue indicates residues shared among species and black the residues that are unique. (TIF) [file pone.0108187.s001.tif]

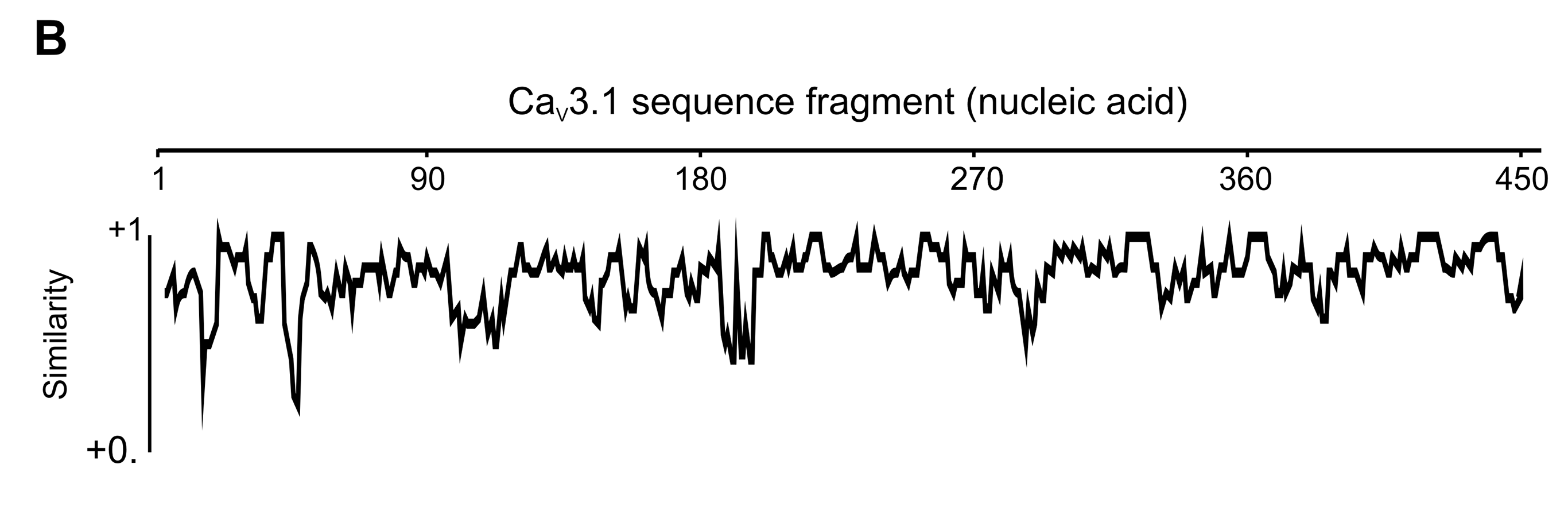

Supplement: Figure S2 — Comparison of the level of similarity among all CaV3.1 sequences shown in Figure S1. (TIF) [file pone.0108187.s002.tif]

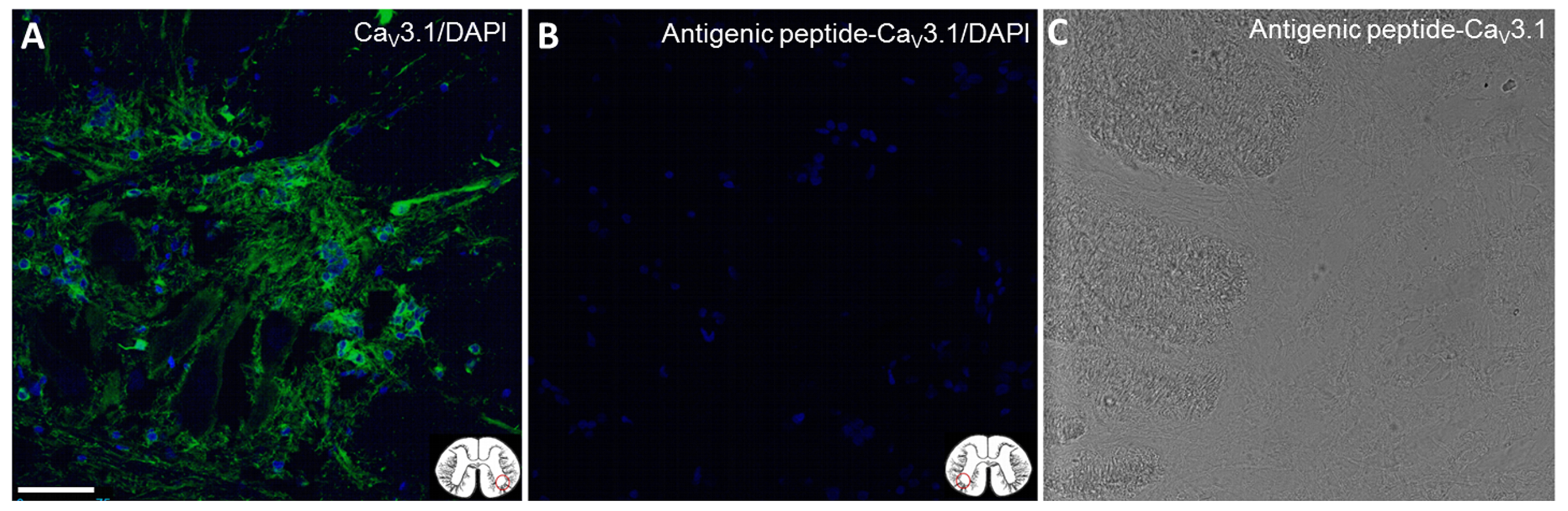

Supplement: Figure S3 — Specificity of the CaV3.1 immunostaining in neurons of the adult turtle spinal cord. Images of transversal sections of the lumbar region are shown. A) Ventral horn CaV3.1 immunoreactivity (green); neuronal nuclei are stained with DAPI (blue). B) The anti-CaV3.1 antibody was pre-incubated with an excess of its antigenic peptide and added to the sample. C) Bright field image of panel B. Scale bar 50 µm. (TIF) [file pone.0108187.s003.tif]
